# Partial Optimization of Endo-1, 4-Β-Xylanase Production by *Aureobasidium pullulans* Using Agro-Industrial Residues

**Published:** 2013-12

**Authors:** Shaghayegh Nasr, Mohammad Reza Soudi, Ali Hatef Salmanian, Parinaz Ghadam

**Affiliations:** 1 National Laboratory of Industrial Microbiology,Department of Biology, Alzahra University, Tehran, Iran; 2 Department of Plant Molecular Biology, National Institute of Genetic Engineering and Biotechnology, Tehran, Iran; 3 Department of Biochemistry, Alzahra University, Tehran, Iran

**Keywords:** *Aureobasidiumpullulans*, Endo-1, 4-β-xylanase, Extracellular enzyme, Optimization

## Abstract

***Objective(s)*** ***: ***Although bacteria and molds are the pioneering microorganisms for production of many enzymes, yet yeasts provide safe and reliable sources of enzymes with applications in food and feed.

***Materials and Methods:*** Single xylanase producer yeast was isolated from plant residues based on formation of transparent halo zones on xylan agar plates. The isolate showed much greater endo-1, 4-β-xylanase activity of 2.73 IU/ml after optimization of the initial extrinsic conditions. It was shown that the strain was also able to produce β-xylosidase (0.179 IU/ml) and α-arabinofuranosidase (0.063 IU/ml). Identification of the isolate was carried out and the endo-1, 4-β-xylanaseproduction by feeding the yeast cells on agro-industrial residues was optimized using one factor at a time approach.

***Results:*** The enzyme producer strain was identified as *Aureobasidiumpullulans. *Based on the optimization approach, an incubation time of 48 hr at 27°C, inoculum size of 2% (v/v), initial pH value of 4 and agitation rate of 90 rpm were found to be the optimal conditions for achieving maximum yield of the enzyme. Xylan, containing agricultural residues, was evaluated as low-cost alternative carbon source for production of xylanolytic enzymes. The production of xylanase enzyme in media containing wheat bran as the sole carbon source was very similar to that of the medium containing pure beechwoodxylan.

***Conclusion:***This finding indicates the feasibility of growing of *A. pullulans* strain SN090 on wheat bran as an alternate economical substrate in order for reducing the costs of enzyme production and using this fortified agro-industrial byproduct in formulation of animal feed.

## Introduction

Hemicellulose is composed of xylan as a major component that constitutes about 20-40% of total plant biomass and accounts for approximately one-third of all renewable organic carbon on earth (1, 2). Xylan is a heteroglycan having a backbone made up of β-1, 4-linked D-xylopyranose residues with substitutions of L-arabinofuranose, D-glucuronic acid and 4-O-methyl-D-glucuronic acid at 2´ and 3´ positions (3). It represents an immense resource of biopolymers for practical applications accounting for 25-30% of the dry biomass of woody tissues of dicots and lignified tissues of monocots and occurs up to 50% in some tissues of cereal grains (4). Complete conversion of hemicellulose requires the action of several main chain and side-chain cleaving enzymes: endoxylanase(endo-1, 4-β-xylanase, E.C.3.2.1.8), β-xylosidase (xylan 1, 4-β-xylosidase, E.C.3.2.1.37), α-glucuronidase (E.C.3.2.1.139), α-arabinofuranosidase (α-L-arabinofuranosidase, E.C.3.2.1.55) and acetyl xylan esterase (E.C.3.1.1.72) (4, 5). Xylanases have been reported to be produced by many microorganisms including fungi and bacteria. Based on the similarities of amino acid sequences, majority of endoxylanases fall in glycoside hydrolase (GH) families 10 and 11, whereas other xylanases are classified in GH families 5, 8 and 43 (6). Xylanases have considerable potential for several industrial applications, e.g. production of pulp and papers, animal feed, food and drinks, textiles and biofuels (7).

The industrial enzyme production is frequently limited by the cost of substrates required for the propagation of producer microorganisms and production of target enzymes. The use of low cost substrates such as agricultural wastes has been suggested as an alternative to reduce the production costs. Agricultural and agro-industrial wastes such as sugarcane bagasse, wheat bran, rice peel, corn straw and corncob, effluents from paper industry, fruit peels and seeds have increased in staggering quantities all over the world as a result of industrialization, becoming a problem, regarding space for disposal and causing environmental pollution. However, the saccharification of those residues which primarily consist of cellulose and hemicelluloses by microbial cellulolytic and xylanolytic enzymes represent an alternative source for the microbial growth aiming the production of biomass or enzymes (8-10). Therefore, as a part of biological solution, the screening of xylanase-producing strains and separation and purification of xylanases are currently of great interest (11, 12).

Reducing the cost of enzyme production may also be possible by optimizing the fermentation medium and other external factors of the process. This is the goal of basic research for industrial applications since the composition of these factors can considerably affect yield of product (13-15). In general, no defined medium has been established for the maximum production of any metabolite or enzyme, because the genetic diversity present in different microbial sources causes each organism or strain to have its own special demands for maximum yield of production (16).

The present work describes successful optimization of fermentation parameters for the production of xylanase by a local and newly isolated yeast-like microorganism,*Aureobasidiumpullulans* strain SN090, using agro-industrial residues.

## Materials and Methods

***Microorganism*** ***isolation***

The microorganism was isolated on YPG medium supplemented with 40 µg/ml of streptomycin and 13µg/ml penicillin (17). The origin of the specimen was epiphytic microorganisms on fruit of a sample of *Capsicum annum* collected from a farm in Alborz Province of Iran. The ability to produce xylanase was qualitatively confirmed when it formed transparent halo zones around colonies on beechwoodxylan agar plates on treatment with congo red followed by washing with 1M NaCl (18). Determination of the extracellular activity of xylanase was qualitatively carried out using cup plate method, described by Hislop*et al*, 1982 (19). The basal medium was prepared using 2% (w/v) agar and 1% (w/v) beechwoodxylan, and pH of the medium was adjusted at 5.6. Three cups were made in each of the agar plate immediately after solidification of the medium. The cups were carefully filled with 100 µl of supernatant by centrifugation of culture broth at 19650 × g for 30 min. The plates were incubated at 30°C for 24 hr and stained as above. 

The stock cultures were maintained on YPG agar slants at 4°C and subcultured at monthly intervals.

***Identification*** ***of the microorganism***

The strain was characterized morphologically, biochemically and physiologically according to standard methods (20, 21). The D_1_/D_2_domain of the 26S rRNAgene was PCR amplified using the primers NL1 and NL4 (22). Sequences of closely related taxa were retrieved and aligned using CLUSTAL W program (23). A phylogenetic tree was constructed by the neighbor-joining method (24). Confidence limits were estimated by bootstrap analysis (1000 replicates) and values of 50% or greater were recorded on the resulting tree only (25).

***Enzymes assay***

Endoxylanase (endo-1, 4-β-xylanase, E.C.3.2.1.8) activity was determined by measuring the release of reducing sugar from a reaction mixture containing 500 µl supernatant and 250 µl of 0.6% xylan aqueous solution followed by addition of 250 µl distilled water and incubation at 30°C for 30 min. The amounts of reducing sugar level in the supernatant were determined by dinitrosalicylic acid (DNS) method (26). β-xylosidase (xylan 1,4–β-xylosidase, E.C.3.2.1.37) and α-arabinofuranosidase (α-L-arabinofuranosidase, E.C.3.2.1.55) activities were assayed using p-nitrophenol-β-xylopyranoside (5 mM) and p-nitrophenol-α-arabinofuranosid (2 mM) as substrates, respectively (27).

Specific activity was measured as the ratio of enzyme activity to protein concentration. Xylanase activity was measured as previously described and protein concentration was quantified by the Bradford method (28) using bovine serum albumin, fraction V (Sigma, USA) as the standard protein.

***Xylanase production***

Submerged fermentation was carried out in 500 ml baffled erlenmeyer flasks containing 100 ml of sterile basal medium contained per liter: Xylan (10 g), (NH_4_)_2_SO_4_ (1.4 g),KH_2_PO_4_ (2.0 g), urea (0.1 g), MgSO_4_.7H_2_O (0.3 g), CaCl_2 _(0.3 g), FeSO_4_.7H_2_O (5.0 mg), MnSO_4_.H_2_O (1.56 mg), CoCl_2 (_2.0 mg) and ZnSO_4_.7H_2_O (1.4 mg) (29). In all experiments, organic and inorganic nitrogen sources in the composition of the basal medium were similar, otherwise mentioned.

***Parametric optimization of xylanase production***

This conventional one-factor-at-a-time approach was carried out in 500 ml flask containing 100 ml basal medium, pH 6 at 30°C, inoculated with 2% (v/v) of fresh culture and shook on a shaking incubator at 150 rpm. In this approach, various nutritional and physical parameters were optimized by maintaining all factors at a constant level in the basal medium, excluding the one under study. Each subsequentfactorwasexaminedaftertakinginto account the previously optimized factors (32). 

**Figure 1. A):**
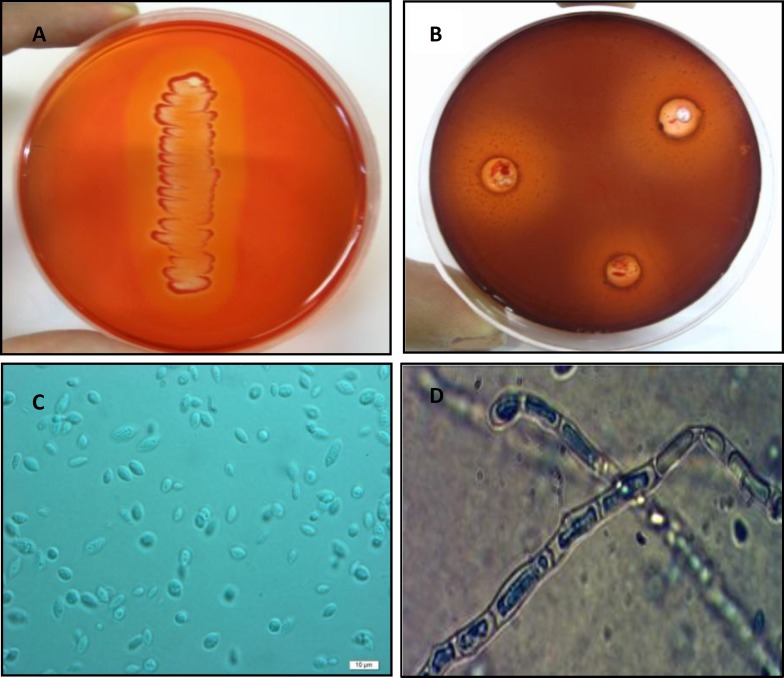
Digestion halos on beechwoodxylan plates indicating xylanase activity **B)** extracellular enzyme production of *Aureobasidiumpullulans* strain SN090 by cup plate method **C)** The yeast cells grown for 48 hr on YPG medium at 30°C** D) **Septatedhyphaegrown on Cornmeal agar at 28°C

The following parameters were evaluated:

- *Incubation period* *:* submerged fermentation was carried out for 24, 48, 72 and 96 hr.
- *Inoculum size* *:* a 24 hr culture (OD 0.50 at 600 nm) at different levels of 0.5, 2, 3.5 and 5 percent (v/v) were used as inocula.

- *Initial* *pH* *:* pH values of the basal medium ranging from 4 to 7 was used for xylanase production. Different pH values were adjusted using normal solutions of caustic soda and hydrochloric acid.

- *Temperature* *:* Incubation of the yeast cultures at different growth temperatures ranging from 24 to 33°C was studied.
- *Agitation:* Shaking speeds ranging from 90 to 180 rpm were used for agitation and aeration of the culture broth.

***Full factorial design of appropriate carbon and nitrogen sources***

A full factorial design was used to investigate the effect of amounts of the most appropriate carbon and nitrogen sources on growth and xylanase production. The grinded carbon sources used in culture medium compositions were wheat bran, rice bran, sugarcane bagasse and beechwoodxylan, each 1% (w/v) concentration.

Effects of organic nitrogen sources (urea, yeast extract,peptone, casamino acids) and inorganic nitrogen sources (ammonium nitrate,ammonium sulfate, ammonium chloride and di-ammonium phosphate) were studied. In every experiment, basal medium was supplemented with final nitrogen concentration of 0.15%. 

All results presented herein are the arithmetic mean of three independent trials.

***Analysis of hydrolyzed substrates***

Enzymatic hydrolysis of xylanwas carried out using 500 µl supernatant of 48 hr incubated culture medium and the same volume of beechwoodxylan (0.6%) plus 1ml of distilled water. The mixture was incubated at 30°C for 30 min and subsequently boiled for 10 min to stop the enzymatic reaction. Afterwards, it was concentrated using rotary vacuum evaporator and 1µl aliquots were spotted on a silica gel F254 chromatography plate (Merck, Germany). Chromatography was developed with a solvent system containing: n-butanol: water: pyridine: toluene (1:6:6:10 v/v). After 4 hr the chromatic plate was air dried and sprayed with Aniline phthalate which was then heated at 100°C for 5 min (30, 31).

## Results

***Isolation and identification of the microorganism***

The local isolate of the yeast-like microorganism, *A. pullulans* strain SN090, was isolated from a sample of *C. annum* collected from Alborz Province of Iran. The isolate showed satisfactory growth on the xylan-containing medium at 30°C. Its ability to produce xylanase enzyme was further confirmed when the related colonies formed orange digestion halos on beechwoodxylan plates after treatment with congo red (Figure 1D). Determination of the extracellular 

**Figure 2:**
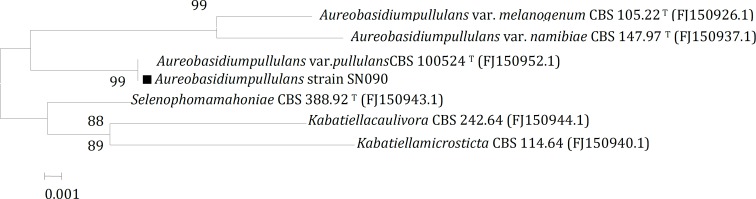
Neighbor-joining tree based on D_1_/D_2_ region of 26S rRNA gene sequence showing the phylogenetic relationships between*Aureobasidiumpullulans* strain SN090 and type strains of related genera. Numbers at branch nodes are bootstrap values (percentages of 1000 replicates). Bar 1 substitution per 1000 nucleotide positions

activity was confirmed with the help of cup-plate method (Figure 1C).

The isolate produced cream to pink colonies on YPG medium and microscopic examination showed undifferentiated cells and frequently-observed budding. Hyphae were hyaline and septate (Figure 1A and 1B). The results of biochemical and physiological characterization of the isolate are shown in Table 1. The sequence of the D_1_/D_2_ domains of the large subunit of rRNA was determined to investigate the relatedness of this strain to other yeast species. Comparing the D_1_/D_2 _sequence of the strain with the closest described species showed a 100% similarity with *Aureobasidium pullulans* var*. pullulans* CBS 584.75. The phylogenetic tree was constructed for strain SN090 as indicated in Figure 2. Therefore, the isolated strain was designated as *A. pullulans* strain SN090 in the NCBI nucleotide sequence databases with an accession number of JF278561.1.

***Enzyme activity***

The yeast cells were grown in liquid medium containing 1% xylan at 30°C, and the culture secreted 2.73 IU/ml of endoxylanase but low levels of β-xylosidase (0.179 IU/ml) and α-L- arabinofuranosidase (0.063 IU/ml).

***Parametric optimization of xylanase production***

**Figure 3:**
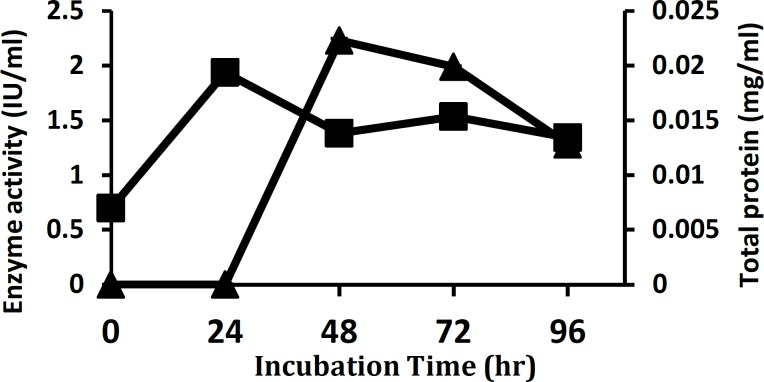
Time course of xylanase production by *Aureobasidium* *pullulans* strain SN090 on wheat bran based media under submerged fermentation. Enzyme activity (▲) and Total protein production (■) are shown. Fermentation parameters are as follows: pH 6, inoculum of 2% (v/v), incubation temperature of 30°C and shaking at 150 rpm

**Figure 4:**
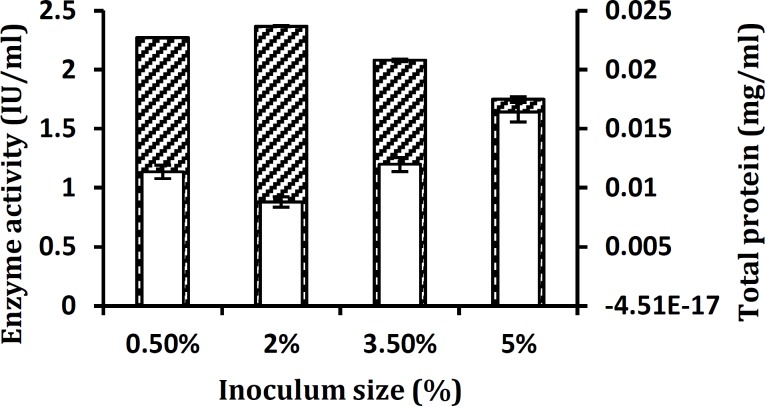
Influence of size of inoculum on xylanase production by *Aureobasidium pullulans* strain SN090 at 30°C under submerged fermentation. Enzyme activity (▲) and total protein production (■) are shown. Fermentation parameters are as follows: pH 6, Incubation time of 48 hr, incubation temperature of 30°C and shaking at 150 rpm

**Figure 5:**
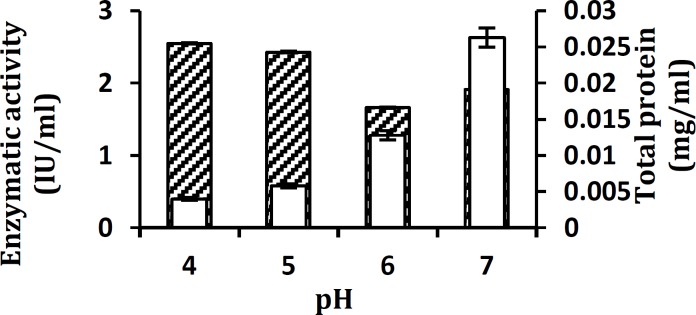
Influence of pH of the medium on xylanase production by *Aureobasidium pullulans* strain SN090 at 30°C under submerged fermentation. Enzyme activity (▲) and total protein production (■) are shown. Fermentation parameters are as follows: Incubation time of 48 hr, inoculum size of 2% (v/v), incubation temperature of 30°C and shaking at 150 rpm

Xylanase activity was the highest (2.23 IU/ml) after 48 hr of incubation and declined on further increasing the time (Figure 3). The effect of inocula on production of xylanase presented in Figure 4, shows that maximum xylanase activity was yielded with 2% (v/v) inoculum and a decrease in xylanase activity was observed at 0.50% (v/v) and higher inocula concentrations of 3.5% and 5% (v/v). The effect of pH values on the production of the extracellular lignocellulose degrading enzyme by *A. pullulans* strain SN090 was investigated in a pH range of 4.0 to 7.0 (Figure 5). The greatest endoxylanase activity (2.55 IU/ml) was detected at the pH level of 4.0.

**Table 1:** Characterization of *Aureobasidium* *pullulans* strain SN090

Character	Reaction	Character	Reaction	Character	Reaction
Assimilation of		Fermentation of		5% NaCl	+
D-Glucose	+	D-Glucose	D	10% NaCl	+
D-Galactose	+	D-Galactose	-	16% NaCl	W
D-Ribose	+	D-Ribose	-	at 25°C	+
D-Xylose	+	D-Xylose	-	at 30°C	+
L-Arabinose	+	L-Arabinose	-	at 35°C	+
D-Arabinose	+	D-Arabinose	-	at 37°C	-
L-Rhamnose	+	L-Rhamnose	-	at 40°C	-
Sucrose	+	Sucrose	+	at 42°C	-
Maltose	+	Maltose	+	0.1% Cycloheximide	-
α-α-Trehalose	+	α-α-Trehalose	+	0.01% Cycloheximide	-
Cellobiose	+	Cellobiose	-	1% acetic acid	-
Salicin	+	Salicin	-	Diazonium Blue B	-
Arbutin	+	Arbutin	-	Urea hydrolysis	+
Melibiose	+	Melibiose	-	Starch formation	-
Lactose	+	Lactose	-	50% Glucose	+
Raffinose	+	Raffinose	D	60% Glucose	+
Melezitose	+	Melezitose	+	Assimilation of	
Inulin	+	Inulin	-	Nitrate	+
Starch	+	Starch	-	Nitrite	+
Ribitol	+	Ribitol	-	Ethylamine	+
Xylitol	+	Xylitol	-	L-Lysine	+
D-Mannitol	+	D-Mannitol	-	Cadaverine	+
Myo-Inositol	+	Myo-Inositol	-	Creatine	+
DL-Lactate	+	DL-Lactate	-	Imidazole	-
Succinate	+	Succinate	-		
D-Gluconic acid	+	D-Gluconic acid	-		
Citrate	-	Citrate	-		
Methanol	-	Methanol	-		
Ethanol	+	Ethanol	-		
					

**Figure 6:**
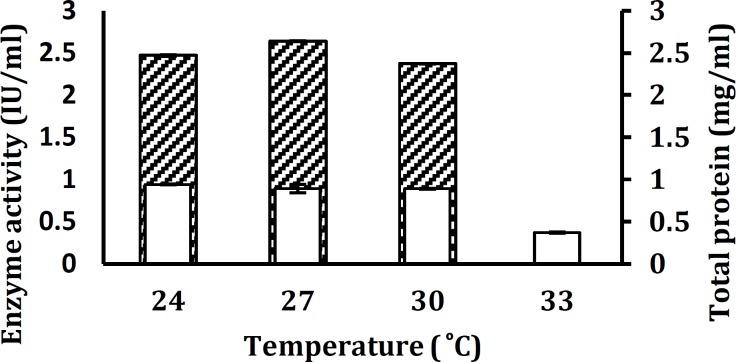
Influence of temperature on xylanase production by *Aureobasidium* *pullulans* strain SN090 under submerged fermentation. Enzyme activity (▲) and Total protein production ( ) are shown. Fermentation parameters are as follows: pH 4, Incubation time of 48 hr, inoculums size of 2% (v/v) and shaking at 150 rpm

**Figure 7:**
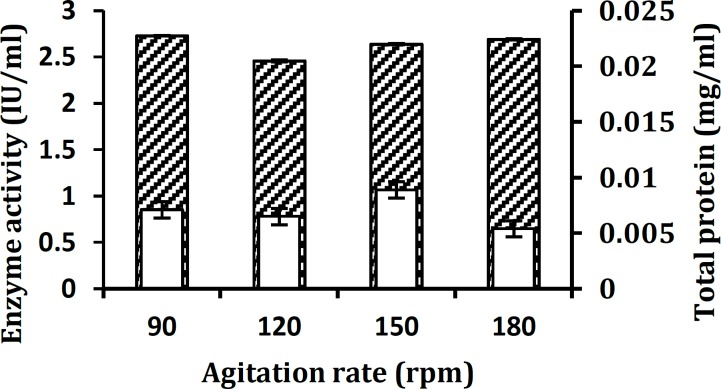
Influence of agitation rate on xylanase production by *Aureobasidium* *pullulans* strain SN090 at 27°C under submerged fermentation. Enzyme activity (▲) and Total protein production ( ) are shown. Fermentation parameters are as follows: pH 4, Incubation time of 48 hr, inoculum size of 2% (v/v), and incubation temperature of 27°C

**Figure 8:**
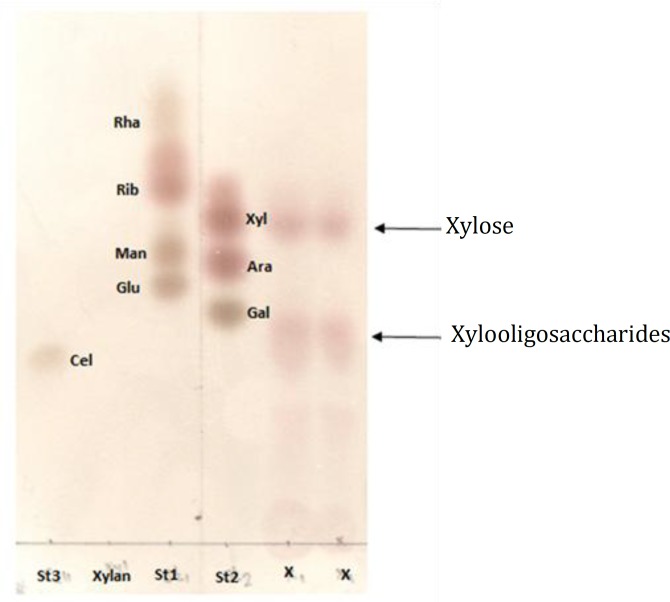
TLC analysis of xylan hydrolyzed products after 30 min incubation at 30°C by crude enzyme. Lane St3: Cel: Cellobiose, Lane Xylan: xylan (supernatant of xylan containing culture broth before inoculation), Lane St1: standard mixture including Glu: Glucose, Man: Mannose, Rib: Ribose, Rha: Rhamnose. Lane St2: standard mixture including Gal: Galactose, Ara: Arabinose, Xyl: Xylose, Lane X: xylan hydrolyzed products

The fermentation temperature appeared to have a dramatic effect on xylanase production. *A. pullulans *strain SN090 produced maximum xylanase activity (2.64 IU/ml) at 27°C, while the lowest productivity (2.37 IU/ml) was at 30°C (Figure 6).

**Table 2:** Effect of combination of carbon and nitrogen sources on xylanase production by*Aureobasidiumpullulans* strain SN090. Fermentation parameters are as follows: pH 4, Incubation time of 48 hr, inoculum size of 2% (v/v), and incubation temperature of 27°C and shaking at 90 rpm

Organic nitrogen sources						
Yeast Extract				Peptone		
Carbon sources	Enzyme activity (iu/ml)	Total protein (mg/ml)	Specific activity (iu/mg)	Enzyme activity (iu/ml)	Total protein (mg/ml)	Specific activity (iu/mg)
Rice bran	0.60±0.08	0.0117±0.0001	51.28	0.92±0.26	0.0107±0.0002	85.98
Wheat bran	2.21±0.06	0.0126±0.0001	175.39	2.13±0.10	0.0126±0.0001	169.04
Sugarcane bagasse	0.91±0.11	0.0091±0.008	100.00	0.51±0.03	0.0056±0.0007	91.07
Beechwoodxylan	1.42±0.08	0.0091±0.0010	156.04	1.42±0.03	0.0057±0.0003	249.12
Casamino acids				Urea		
Carbon sources	Enzyme activity (iu/ml)	Total protein (mg/ml)	Specific activity (iu/mg)	Enzyme activity (iu/ml)	Total protein (mg/ml)	Specific activity (iu/mg)
Rice bran	1.44±0.20	0.0081±0.0009	177.77	0.26±0.05	0.0234±0.0010	11.11
Wheat bran	2.19±0.9	0.0126±0.0004	173.80	1.34±0.02	0.0319±0.0001	42.00
Sugarcane bagasse	1.11±0.12	0.0047±0.0004	236.17	0.43±0.07	0.0098±0.0004	43.87
Beechwoodxylan	2.66±0.04	0.0010±0.0003	2660	1.57±0.11	0.0246±0.0003	63.82
Inorganic nitrogen sources						
Ammonium nitrate				Ammonium sulfate		
Carbon sources	Enzyme activity (iu/ml)	Total protein (mg/ml)	Specific activity (iu/mg)	Enzyme activity (iu/ml)	Total protein (mg/ml)	Specific activity (iu/mg)
Rice bran	0.67±0.17	0.0070±0.0005	95.71	0.36±0.02	0.0115±0.0001	31.30
Wheat bran	2.38±0.29	0.0126±0.0010	188.88	2.54±0.22	0.0152±0.0020	167.10
Sugarcane bagasse	1.56±0.19	0.0033±0.0003	472.72	1.29±0.19	0.0029±0.0002	444.82
Beechwoodxylan	2.33±0.09	0.0020±0.0008	1165	2.21±0.025	0.0009±0	2455.55
Ammonium chloride				Di-ammonium phosphate		
Carbon sources	Enzyme activity (iu/ml)	Total protein (mg/ml)	Specific activity (iu/mg)	Enzyme activity (iu/ml)	Total protein (mg/ml)	Specific activity (iu/mg)
Rice bran	0.38±0.04	0.0075±0.0007	50.66	0.44±0.12	0.0075±0.0007	58.66
Wheat bran	2.35±0.17	0.0129±0.0007	182.17	2.40±0.21	0.0126±0.0001	190.47
Sugarcane bagasse	1.53±0.12	0.0008±0.0010	1912.50	1.30±0.26	0.0040±0.0004	325
Beechwoodxylan	2.72±0.15	0.0014±0.0003	1942.85	1.77±0.11	0.0014±0.0003	1264.28

Values are the arithmetic means of results from triplicate experiments

The effect of optimal agitation rate for xylanase production was obtained after 48 hr of culture in baffle erlenmeyer flasks at different agitation speeds. Data indicated that xylanase production at 90 rpm was higher than other agitation speeds (Figure 7).

The influence of using different sources of carbon as well as organic and inorganic nitrogen sources for xylanase production was assessed and wheat bran supported the appropriate enzyme synthesis (Table 2), whereas the other carbon sources resulted in remarkably lower enzyme production. As shown in Table 2, in the presence of wheat bran as a source of carbon, organic nitrogen sources such as yeast extract, peptone and casamino acids resulted in higher enzyme titers, i.e. 2.21 IU/ml, 2.13 IU/ml, and 2.19 IU/ml, respectively. The lowest level of activity and the highest rate of total protein production were achieved with urea, while inorganic compounds such as ammonium nitrate, ammonium sulfate, di-ammonium phosphate produced 2.38 IU/ml,2.54 IU/ml and 2.4038 IU/ml of xylanase, respectively, in similar conditions.

***Analysis of hydrolyzed products***

Hydrolytic products of xylanwere studied by treating the xylan with crud xylanase, extracted from culture broth at 30°C for 30 min. It can be observed that the main product of xylan hydrolysis was xylose (Figure 8). However, xylooligosaccharides from various degree of polymerization were also present. 

## Discussion

A xylanase-producing yeast-like microorganism was obtained by the screening method of transparent zone on a selective medium of 1% beechwoodxylan. The results of hydrolytic products of xylan suggested that xylanase initially cleaved the substrate to free xylooligosaccharides and then the resulting oligosaccharides were probably cleaved to form xylose.

The isolate was identified as *A. pullulans* using polyphasic approach including morphological analysis, standard physiological profiling and DNA sequencing. The isolate secreted a good amount of endoxylanase (2.73 IU/ml), but low levels of β-xylosidase (0.179 IU/ml) and α-L- arabinofuranosidase (0.063 IU/ml).

Improvement in production of any metabolite by microbial activity depends on physiological, nutritional and biochemical nature of the microbe employed, considering that such factors vary from one organism to another (16). Therefore, one factor at the time methodology was applied for the optimization of xylanase biosynthesis by submerged cultivation of *A. pullulans* strain SN090. In this conventional scaling-up approach, various nutritional and physical parameters were optimized by maintaining all factors at a constant level in the basal medium, except for the one under study. Each subsequent factor was examined after taking into account the previously optimized factors (32). In the first optimization step and once the time course of xylanase production was determined, the maximal activity (2.23 IU/ml) was found after 48 hr of incubation which was occurred during the growth phase of culture. Further incubation up to 72 hr did not show any increment in the levels of enzyme production along with a decrease in the enzyme activity of the supernatant (Figure 3). The phenomenon of sudden increase and subsequent decrease in enzyme activities during the cultivation period has also been noted in xylanase being produced by other microorganisms including other fungus and members of *Streptomyces* spp. (27, 33). It may have been occurred as a result of proteolysis or due to depletion of available nutrients for the isolate, hence causing a stressed microbial physiology that can result in enzyme inactivation (34). It can therefore suggest that the endpoint of fermentation should be carefully controlled since synthesized xylanase could be degraded by non-specific proteases secreted by fungus (14, 33). On the other hand, it is obvious that production costs are directly proportional to productivity of the yeast cells and the production time, so that costs of production time should be considered.

The effect of inocula on production of xylanase was observed. Maximum xylanase activity was yielded with 2% (v/v) inoculum. However, decreased xylanase activity was observed at lower and higher inocula concentrations. It is reported that inoculum size can influence the growth of the organism thus the efficiency of enzyme production. Increased inoculum size may support adequate growth and eventually efficient enzyme production (35).

The pH levels and temperature are important environmental parameters that determine growth rate of the microorganism and has major effect on levels of enzyme production. The highest endoxylanase activity (2.55 IU/ml) was observed at the pH level of 4.0.

The cells of *A. pullulans* strain SN090 was cultivated in baffle erlenmeyer flasks and incubated at different temperatures. Although the physiological changes being induced by high temperatures during enzyme production are not completely understood, it has been suggested that at high temperatures, microorganisms may synthesize only a limited number of proteins essential for growth and other physiological processes (14). Such results indicate that the production of extracellular enzyme involved in lignocellulose degradation was completely growth associated. This is in accordance with a previous study on fungi and *actinomycete *cultures (27). The culture did not show any enzyme production at higher temperature (33°C). These high temperatures can induce enzymes inactivation in metabolic pathways and accumulation of further by-products such as acids from the citric acid cycle which may lower the values of all kinetic parameters while low temperatures may prevent flow of nutrients across the cell membrane which can result in high demand for maintenance energy (36).

The optimal agitation rate for the extracellular xylanase activity was 90 rpm rather than 180 rpm, and the amount of extracellular protein apparently increased at this agitation speed (Figure 7). This was probably because of the deleterious effect of high shear stress on the fungus that was perhaps due to the higher sheering force, which caused cell lyses and release of proteases that can inhibit xylanase production (37).

Selecting an appropriate substrate is of great importance for successful production of xylanases. The substrates not only serve as carbon and energy sources, but also provide the necessary inducing compounds for better growth and improved enzyme production by the organism, preferentially for an extended period of cultivation. 

Xylan, as the substrate, is costly for large-scale production of xylanases; however, lignocellulosic materials can be used as cost-effective substrates for this purpose (38, 39).Among the lignocellulosic materials tested as carbon sources in this research, wheat bran was far more effective for xylanase production, whereas the other carbon sources resulted in markedly lower activities. The considerable difference in xylanase titers when 1% wheat bran was used as the main carbon source, may be attributed to its content of hemicellulose material, favorable degradability and the presence of some other nutrients within the substrate (39, 40). Wheat bran, as a by-product of wheat flour processing, accounts for 15-20% of the grain weight and contains 20-30% arabinoxylan (41). Moreover, it contains considerable amounts of soluble sugars required for the microorganism growth (42). Accordingly, inclusion of this plant residue in the nutrient medium composition has an exceptionally important economical significance in xylanase production. As shown in our research, using organic and inorganic nitrogen sources fluctuantions in xylanase activities were observed and results showed inconsistency, whereas inorganic compounds generally induced more xylanase production than the organic ones. These differences in enzyme activity being obtained from cultivated media containing various complex nitrogen sources could be caused by their varying contents of aminoacids, peptides, vitamins, trace elements and/or mineral salts. Among organic compounds, casamino acids clearly gave the best results for the production of xylanase activity. Lowest activity level and highest rate of total protein production was achieved with urea, a recalcitrant source of protein, and resulted in strong repression of xylanase biosynthesis.

## Conclusion

The results of this study would be of significance for agricultural and enzyme industries to develop innovative techniques of utilizing wheat bran as an inexpensive agro-industrial waste for enzyme production, which will facilitate the large-scale, economically viable production processes of xylanase. Nevertheless, the xylanase production by this strain is relatively low. Genetic improvements should be experimented in order to increase the level of xylanase production of this strain.
